# Synthesis of new C^α^-tetrasubstituted α-amino acids

**DOI:** 10.3762/bjoc.5.5

**Published:** 2009-02-18

**Authors:** Andreas A Grauer, Burkhard König

**Affiliations:** 1 Institute for Organic Chemistry, University of Regensburg, Universitätsstrasse 31, 93040 Regensburg, Germany

**Keywords:** amino acid synthesis, C^α^-tetrasubstituted α-amino acids, unnatural amino acids

## Abstract

C^α^-Tetrasubstituted α-amino acids are important building blocks for the synthesis of peptidemimetics with stabilized secondary structure, because of their ability to rigidify the peptide backbone. Recently our group reported a new class of cyclic C^α^-tetrasubstituted tetrahydrofuran α-amino acids prepared from methionine and aromatic aldehydes. We now report the extension of this methodology to aliphatic aldehydes. Although such aldehydes are prone to give aldol products under the reaction conditions used, we were able to obtain the target cyclic amino acids in low to moderate yields and in some cases with good diastereoselectivity.

## Introduction

The secondary structure of an amino acid sequence is of key importance for the biological activity of proteins [1–2]. In most cases short natural peptide sequences containing L α-amino acids are very flexible and do not show a distinct secondary structure in aqueous solution. Therefore small oligopeptides of natural amino acids are often not suitable for structural studies, as drug candidates or as biological probes. One way to restrict the conformation and introduce rigidity is the use of C^α^-tetrasubstituted α-amino acids, in which the α-hydrogen atom of the α-amino acid is replaced by an alkyl or aryl group [3–4]. These building blocks if compared to natural α-amino acids show more constrained conformations and are stereochemically stable due to the quaternary carbon centre [5]. By the use of such amino acids, even short peptide sequences can be made to adopt stable secondary structures like β-turns [6], or 3_10_- or α-helices [7–11]. C^α^-Tetrasubstituted α-amino acids are therefore of importance for the synthesis of peptides or peptidomimetics with properties such as increased chemical and metabolic stability, increased hydrophobicity, or increased conformational constraints [12–14]. Therefore a variety of C^α^-tetrasubstituted α-amino acids have been developed in recent years and reported in the literature [15–19].

Recently our group reported as a new class of such unnatural amino acids C^α^-tetrasubstituted tetrahydrofuran amino acids (TAAs), which were prepared from commercially available racemic methionine in a four step synthesis. TAAs show the ability to induce stable β-turns in solid state and solution when incorporated into natural peptide sequences [20].

**Figure 1:**
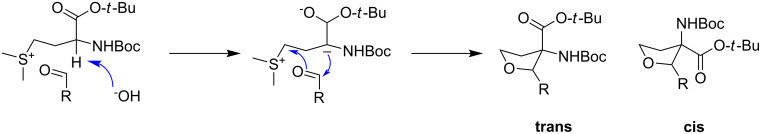
Proposed reaction mechanism for the formation of C^α^-tetrasubstituted tetrahydrofuran α-amino acids.

The key step of the TAA synthesis is the ring formation, which is an aldol type reaction between the sulfonium salt **1** and an aldehyde. Applying strongly basic conditions leads to the abstraction of the α-proton of the methionine, the aldehyde undergoes nucleophilic attack by the ester enolate and the displacement of the sulfonium salt substituent by the alkoxy group gives the target TAA. To avoid undesired side reactions under the basic reaction conditions only aldehydes which lack protons in the α-position like benzaldehyde derivatives were used until now (see Figure 1).

We report here the synthesis and structural characterization of cyclic tetrahydrofuran C^α^-tetrasubstituted amino acids which were prepared from aliphatic aldehydes.

## Results and Discussion

**Scheme 1:**
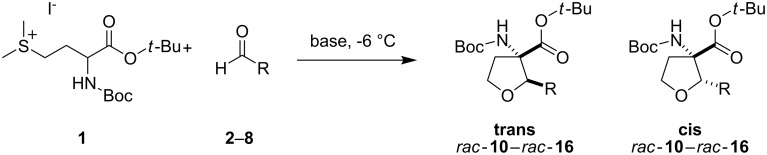
Cyclisation reaction forming the tetrahydrofuran amino acid (TAA).

The major difference between the previously used aromatic aldehydes and their aliphatic counterparts is their ability to undergo an aldol reaction under the strongly basic conditions necessary for the formation of the tetrahydrofuran ring system (see Scheme 1).

Therefore different bases (namely KOH, KO*t*-Bu and CsOH) and also different equivalents of sulfonium salt **1**, aldehyde and base were screened to find the optimal conditions for the TAA synthesis. In all cases, dry acetonitrile was used as solvent, and the temperature was kept at −6 °C to improve the diastereoselectivity of the ring formation. *n*-Butyraldehyde (**2**) was chosen for the first set of reactions to optimize the reaction conditions. Table 1 summarizes the results. In total eight different reaction conditions were tried and the conversion was determined by ^1^H NMR analysis after aqueous workup. Entries 1–3 clearly indicate that caesium hydroxide is the base of choice for the conversion of aliphatic aldehydes, which is in contrast to the reactions carried out with aromatic aldehydes where KOH gave the best results [20]. In the next three experiments (entries 4–6), the amount of sulfonium salt **1** was increased with respect to the aldehyde and the optimal amount of base was investigated. The highest yields of TAA were obtained with identical amounts of base and sulfonium salt. To optimize the ratio of aldehyde to sulfonium salt **1**, reactions with ratios of 1:1.5, 2:1 and 8:1 aldehyde to sulfonium salt **1** were performed (entries 6–8). Best results were obtained with a slight excess of the sulfonium salt.

**Table 1:** Results of the reaction condition optimization of the reaction between the sulfonium salt **1** and *n*-butyraldehyde (**2**)

Entry	equiv sulfonium salt **1**^a^	equiv base^a^			Yield^b^
		KOH	KO*t*-Bu	CsOH	

1	1.1	1.1			16%^c^
2	1.1		1.1		25%^c^
3	1.1			1.1	32%^c^
4	1.5			1.0	21%^c^
5	1.5			1.25	15%^c^
6	1.5			1.5	47%^c^
7	0.5			0.5	24%^d^
8	0.125			0.125	13%^d^

All reactions were performed following GP1. ^a^Equivalents are with respect to *n*-butyraldehyde (**2**). ^b^Yields were determined by ^1^H NMR and have an error of ± 5% as estimated from repeating the reaction of entry 6. ^c^Yields are based on the aldehyde as limiting compound. ^d^Yields are based on the sulfonium salt as limiting compound.

To explore the scope of the reaction a series of aliphatic aldehydes was converted using the optimized reaction conditions. The results are summarized in Table 2 and show that sterically demanding aliphatic aldehydes like pivalaldehyde (**3**), which has a tertiary α-carbon, do not undergo the ring closing reaction. Aldehydes with a secondary α-carbon like 2-phenylpropanal (**4**) or isobutyraldehyde (**5**, entries 2 and 3) fail or form the product only in traces. 3-Methylbutanal (**6**, entry 4), which bears a secondary carbon atom in the β-position, gives increased product yields. The best results were obtained for the unbranched *n*-butyraldehyde (**2**) and acetaldehyde (**7**) with yields of 36% and 28%, respectively, (entries 5 and 6).

**Table 2:** Scope of the reaction of sulfonium salt **1** with different aldehydes or ketones.

Entry	Aldehyde or ketone	Product	Isolated yield (%)	*trans*/*cis*-Ratio^a^

1	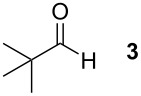	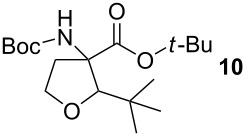	–	–
2	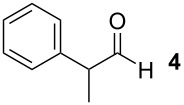	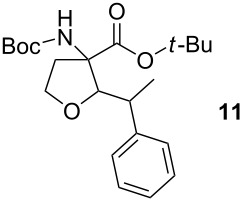	–	–
3	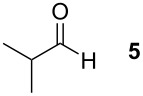	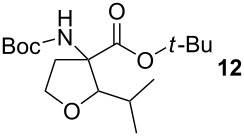	2	9/1
4	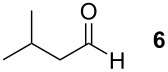	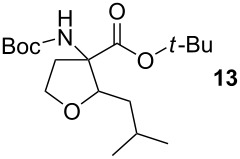	17	5/1
5	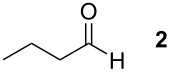	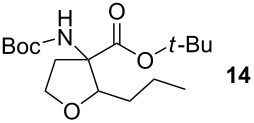	36	3/1
6	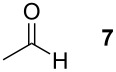	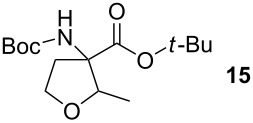	28	2/1
7	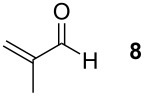	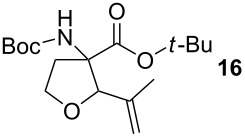	18	>95/5
8	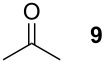	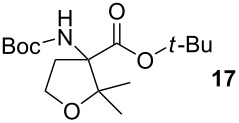	–	–

^a^The *trans*/*cis*-ratio was determined by NMR spectroscopy.

Methacrylaldehyde (**8**, entry 7) as an example of an unsaturated aldehyde was reacted with the sulfonium salt **1**. The corresponding TAA was obtained in 18% yield. To investigate the reactivity of ketones, acetone (**9**) was tested but no TAA product was formed.

**Figure 2:**
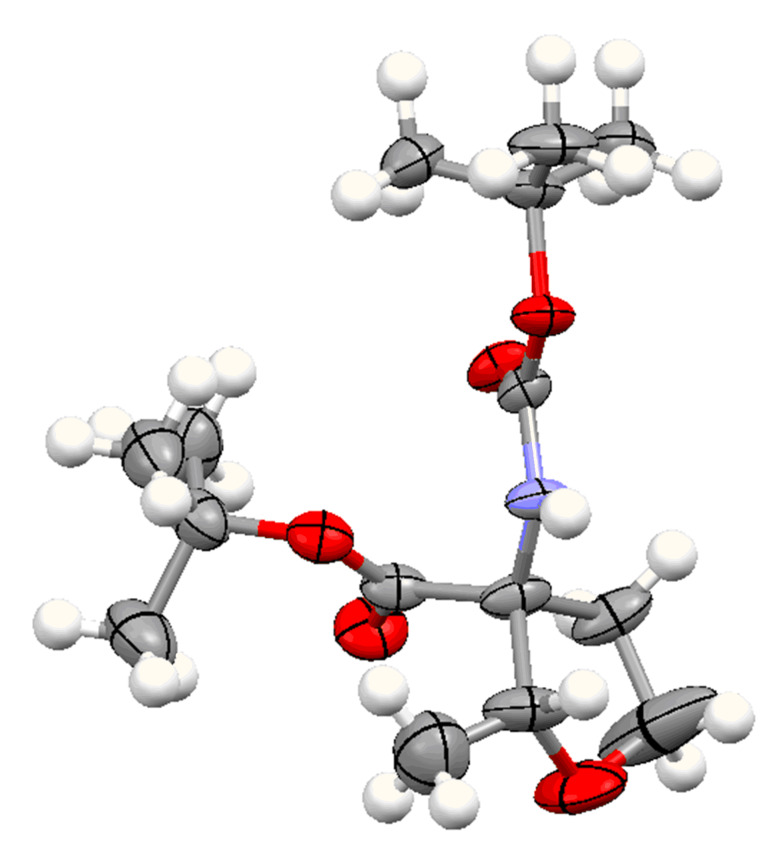
Structure of compound **15** in the solid state determined by X-ray analysis [21].

The diastereoselectivity of the reaction was also examined. The previously reported ring formation reactions with aromatic aldehydes showed *trans*-selectivity [20]. Therefore it was assumed that the major product with aliphatic aldehydes is also *trans* configured. To prove this assumption 2-dimensional NMR spectroscopy was performed. The crystal structure obtained from compound **15** shows the *trans*-diastereomer (see Figure 2). In the *trans*-product the proton of the amine and the proton of the THF-CH group are located on the same side of the ring and are therefore in close proximity with a distance determined from the crystal structure of 2.18 Å. This close distance results in a strong NOE cross peak, while for the *cis*-product in which the two protons point to opposite sides of the ring no NOE cross peak occurs. The occurrence of the NOE cross peak confirms that the main diastereomer is *trans* configured.

The diastereoselectivity of the reaction varies. For acetaldehyde (**7**) a ratio of to 2/1 in favour of the *trans*-product was found. This ratio increases to 3/1 (*trans*/*cis*) for the larger *n*-butyraldehyde (**2**). With increased steric demand of the aldehydes the ratio shifts in favour of the *trans*-product as for the 3-methylbutanal (**6**, *trans*/*cis* = 5/1) and for isobutyraldehyde (**5**, *trans*/*cis* = 9:1). The highest *trans*-selectivity was found for methacrylaldehyde (**8**) where no *cis*-product could be detected by NMR.

## Conclusion

C^α^-Tetrasubstituted tetrahydrofuran α-amino acids can be obtained from aliphatic aldehydes in low to moderate yields. The diastereoselectivity of the reaction depends on the steric demand of the aldehyde and varies from *trans*/*cis*-ratios of 2/1 up to 95/5. Although the scope of the reported reaction is certainly limited, the study shows that from selected aliphatic aldehydes C^α^-tetrasubstituted tetrahydrofuran α-amino acids are accessible in synthetically useful yields and selectivities.

## Experimental

All reagents and solvents used were of analytical grade purchased from commercial sources and were used without further purification if not stated otherwise. All reactions were carried out under an atmosphere of nitrogen. Acetonitrile was dried prior to use. All aldehydes were distilled shortly before use. Silica gel 60 (230–400 mesh) was used for column chromatography. TLC was carried out with silica gel 60 F_254_ plates. Visualization was accomplished by UV light and ninhydrin staining. All NMR spectra were recorded on a Bruker Avance 300 (300 MHz) instrument with external standard. For NMR spectra of all new compounds see Supporting Information File 1.

The sulfonium salt **1** was synthesized according to a known literature procedure [20].

### General Procedure for the tetrahydrofuran amino acid synthesis (GP 1)

In an oven dried Schlenk flask under nitrogen atmosphere the sulfonium salt (0.125–1.5 equiv with respect to the aldehyde) was dissolved in dry acetonitrile (5 ml per 1 mmol sulfonium salt). The colourless solution was cooled to −6 °C and the base (0.125–1.5 equiv with respect to the aldehyde) followed by the freshly distilled aldehyde were added. The mixture was stirred at −6 °C for 4 h. After complete consumption of starting material the reaction mixture was quenched with water (4 ml per mmol sulfonium salt) and was extracted with diethyl ether (1 × 4 ml/mmol, 2 × 5 ml/mmol sulfonium salt). The combined organic layers were washed with brine solution and dried over MgSO_4_. After removal of the solvent under reduced pressure the crude product was purified by flash chromatography using mixtures of diethyl ether and petrol ether (PE) (15:85 → 20:80) as eluent.

### *tert*-Butyl 3-(*tert*-butoxycarbonylamino)-2-isopropyltetrahydrofuran-3-carboxylate (**12**)

The synthesis followed GP 1 using [3-(*tert*-butoxycarbonylamino)-4-*tert*-butoxy-4-oxobutyl]dimethylsulfonium iodide (**1**, 1.25 g, 2.79 mmol, 1.5 equiv), caesium hydroxide (418 mg, 2.79 mmol, 1.5 equiv) and isobutyraldehyde (**5**, 169 µl, 1.86 mmol, 1 equiv). The product was purified with a 85:15 mixture of PE:diethyl ether (*R_f_* = 0.2) to give **12** as a colourless oil in 2% yield (18 mg, 0.05 mmol). The product was obtained as an inseparable mixture of the *cis* and *trans* product with a *cis*:*trans* ratio of 1:9. ^1^H NMR (CDCl_3_): δ = 0.90–0.92 (m, 6 H, CH_3_); 1.41–1.52 (m, 18 H, *t*-Bu-CH_3_); 1.69–1.80 (m, 1 H, CH); 2.42 (bs, 1 H, CH_2_); 2.62–2.79 (m, 1 H, CH_2_); 3.45–3.55 (m, 1 H, CH_2_); 3.88–4.11 (m, 2 H, CH_2_ + CH); 5.02 (bs, 0.1 H, *cis*-NH); 5.28 (bs, 0.9 H, *trans*-NH). ^13^C NMR (CDCl_3_): δ 16.7 (+, 0.1 C, *cis*-CH_3_); 17.2 (+, 0.9 C, *trans*-CH_3_); 18.5 (+, 0.1 C, *cis*-CH_3_); 19.1 (+, 0.9 C, *trans*-CH_3_); 20.4 (+, 0.1 C, *cis*-CH); 20.8 (+, 0.9 C, *trans*-CH); 27.9 (+, 3 C, *t*-Bu-CH_3_); 28.4 (+, 3 C, *t*-Bu-CH_3_); 32.6 (−, 1 C, CH_2_); 66.5 (−, 0.1 C, *cis*-OCH_2_); 66.6 (−, 0.9 C, *trans*-OCH_2_); 67.6 (C_quat_, 1 C, NHC); 82.3 (C_quat_, 2 C, *t*-Bu-C); 87.5 (+, 1 C, CH); 154.0 (C_quat_, 0.1 C, *cis*-NHCO); 154.4 (C_quat_, 0.9 C, *trans*-NHCO); 171.2 (C_quat_, 0.1 C, *cis*-COO); 171.3 (C_quat_, 0.9 C, *trans*-COO). IR (neat) [cm^−1^]: 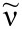 = 2977, 2877, 2361, 1708, 1492, 1392, 1366, 1250, 1157, 1085, 1052, 940, 848. CI-MS (NH_3_): *m/z* 218.2 (9) [MH^+^ − 2 C_4_H_8_], 274.2 (51) [MH^+^ − C_4_H_8_], 330.2 (100) [MH^+^]. HR-MS (FAB, MeOH/glycerol) [MH^+^] calcd. for C_17_H_32_NO_5_ 330.2280; found 330.2288. MF C_17_H_31_NO_5_. MW 329.43.

### *tert*-Butyl 3-(*tert*-butoxycarbonylamino)-2-isobutyltetrahydrofuran-3-carboxylate (**13**)

The synthesis followed GP 1 using [3-(*tert*-butoxycarbonylamino)-4-*tert*-butoxy-4-oxobutyl]dimethylsulfonium iodide (**1**, 1.25 g, 2.79 mmol, 1.5 equiv), caesium hydroxide (418 mg, 2.79 mmol, 1.5 equiv) and 3-methylbutanal (**6**, 201 µl, 1.86 mmol, 1 equiv). The product was purified with a 85:15 mixture of PE:diethyl ether (*R_f_* = 0.16) to give **13** as a colourless oil in 17% yield (106 mg, 0.18 mmol). The product was obtained as an inseparable mixture of the *cis* and *trans* product with a *cis*:*trans* ratio of 1:5. ^1^H NMR (CDCl_3_): δ 0.87–0.90 (m, 6 H, CH_3_); 1.30–1.41 (m, 2 H, CH_2_); 1.44–1.47 (m, 18 H, *t*-Bu-CH_3_); 1.71–1.80 (m, 1 H, CH); 2.37 (bs, 1 H, CH_2_); 2.65–2.85 (m, 1 H, CH_2_); 3.76–4.08 (m, 3 H, CH_2_ + CH); 4.93 (bs, 0.2 H, *cis*-NH); 5.12 (bs, 0.8 H, *trans*-NH). ^13^C NMR (CDCl_3_): δ 21.6 (+, 0.8 C, *trans*-CH_3_); 21.9 (+, 0.2 C, *cis*-CH_3_); 23.6 (+, 0.8 C, *trans*-CH_3_); 23.7 (+, 0.2 C, *cis*-CH_3_); 25.2 (+, 0.8 C, *trans*-CH); 25.7 (+, 0.2 C, *cis*-CH); 27.9 (+, 3 C, *t*-Bu-CH_3_); 28.3 (+, 3 C, *t*-Bu-CH_3_); 38.0 (−, 0.2 C, *cis*-CH_2_); 39.3 (−, 0.8, *trans*-CH_2_); 66.3 (−, 0.2 C, *cis*-OCH_2_); 66.9 (−, 0.8 C, *trans*-OCH_2_); 67.6 (C_quat_, 0.2 C, *cis*-NHC); 68.2 (C_quat_, 0.8 C, *trans*-NHC); 79.8 (C_quat_, 1 C, *t*-Bu-C); 81.7 (C_quat_, 0.2 C, *cis*- *t*-Bu-C); 82.0 (C_quat_, 0.8 C, *trans*- *t*-Bu-C); 82.1 (+, 0.8 C, *trans*-CH); 82.8 (+, 0.2 C, *cis*-CH); 154.7 (C_quat_, 0.8 C, *trans*-NHCO); 155.1 (C_quat_, 0.2 C, *cis*-NHCO); 170.6 (C_quat_, 0.2 C, *cis*-COO); 170.7 (C_quat_, 0.8 C, *trans*-COO). IR (neat) [cm^−1^]: 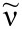 = 3326, 2957, 2359, 1708, 1497, 1366, 1250,1160, 1116, 1098, 1055, 885, 849, 781. CI-MS (NH_3_): *m/z* (%) = 244.2 (25) [MH^+^ − Boc], 288.2 (44) [MH^+^ − C_4_H_8_], 305.2 (19) [MNH_4_^+^ − C_4_H_8_], 344.3 (100) [MH^+^]. HR-MS (FAB, MeOH/glycerol): [M^+^] calcd. for C_18_H_33_NO_5_ 343.2359; found 343.2358. MF C_18_H_33_NO_5_. MW 343.46.

### *tert*-Butyl 3-(*tert*-butoxycarbonylamino)-2-propyltetrahydrofuran-3-carboxylate (**14**)

The synthesis followed GP 1 using [3-(*tert*-butoxycarbonylamino)-4-*tert*-butoxy-4-oxobutyl]dimethylsulfonium iodide (**1**, 480 mg, 0.75 mmol, 1.5 equiv), caesium hydroxide (112 mg, 0.75 mmol, 1.5 equiv) and butyraldehyde (**2**, 44 µl, 0.5 mmol, 1 equiv). The product was purified with a 85:15 mixture of PE:diethyl ether (*R_f_* = 0.21) to give **14** as a colourless oil in 36% yield (60 mg, 0.18 mmol). The product was obtained as an inseparable mixture of the *cis* and *trans* product with a *cis*:*trans* ratio of 1:3. ^1^H NMR (CDCl_3_): δ 0.86 (q, ^3^J_H,H_ = 8.2, 3 H, CH_3_); 1.21–1.55 (m, 22 H, *t*-Bu-CH_3_ + CH_2_); 2.29 (bs, 1 H, CH_2_); 2.61–2.79 (m, 1 H, CH_2_); 3.62–4.15 (m, 3 H, CH_2_ + CH); 4.91 (bs, 0.25 H, *cis*-NH); 5.01 (bs, 0.75 H, *trans*-NH). ^13^C NMR (CDCl_3_): δ 14.0 (+, 0.75 C, *trans*-CH_3_); 14.1 (+, 0.25 C, *cis*-CH_3_); 19.8 (−, 0.75 C, *trans*-CH_2_); 20.0 (−, 0.25 C, *cis*-CH_2_); 27.9 (+, 3 C, *t*-Bu-CH_3_); 28.3 (+, 3 C, *t*-Bu-CH_3_); 31.3 (−, 0.25 C, *cis*-CH_2_); 32.7 (−, 0.75 C, *trans*-CH_2_); 35.6 (−, 0.25 C, *cis*-CH_2_); 37.2 (−, 0.75 C, *trans*-CH_2_); 66.2 (−, 0.25 C, *cis*-OCH_2_); 66.8 (−, 0.75 C, *trans*-OCH_2_); 67.3 (C_quat_, 0.25 C, *cis*-NHC); 68.2 (C_quat_, 0.75 C, *trans*-NHC); 79.8 (C_quat_, 1 C, *t*-Bu-C); 81.7 (C_quat_, 0.25 C, *cis*- *t*-Bu-C); 82.0 (C_quat_, 0.75 C, *trans*- *t*-Bu-C); 84.1 (+, 0.75 C, *trans*-CH); 84.5 (+, 0.25 C, *cis*-CH); 154.8 (C_quat_, 0.25 C, *cis*-NHCO); 155.1 (C_quat_, 0.75 C, *trans*-NHCO); 170.6 (C_quat_, 0.75 C, *trans*-COO); 170.7 (C_quat_, 0.25 C, *cis*-COO). IR (neat) [cm^−1^]: 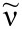 = 3334, 2975, 2357, 1709, 1490, 1366, 1250, 1157, 1077, 949, 848. CI-MS (NH_3_): *m/z* (%) = 230.2 (18) [MH^+^ − Boc], 274.2 (30) [MH^+^ − C_4_H_8_], 291.2 (19) [MNH_4_^+^ − C_4_H_8_], 330.2 (100) [MH^+^], 676.6 (9) [2 M + NH_4_^+^]. HR-MS (FAB, MeOH/glycerol): [M^+^] calcd. for C_17_H_31_NO_5_ 329.2202; found 329.2210. MF C_17_H_31_NO_5_. MW 329.43.

### *tert*-Butyl 3-(*tert*-butoxycarbonylamino)-2-methyltetrahydrofuran-3-carboxylate (**15**)

The synthesis followed GP 1 using [3-(*tert*-butoxycarbonylamino)-4-*tert*-butoxy-4-oxobutyl]dimethylsulfonium iodide (**1**, 670 mg, 1.50 mmol, 1.2 equiv), caesium hydroxide (225 mg, 1.50 mmol, 1.2 equiv) and acetaldehyde (**7**, 70 µl, 1.25 mmol, 1 equiv). The product was purified with a 80:20 mixture of PE:diethyl ether (*R_f_* = 0.1) to give **15** as a colourless oil in 28% yield (105 mg, 0.93 mmol). The product was obtained as an inseparable mixture of the *cis* and *trans* product with a *cis*:*trans* ratio of 1:2. ^1^H NMR (CDCl_3_): δ 1.16 (d, ^3^J_H,H_ = 6.3, 2 H, *trans*-CH_3_); 1.23 (d, ^3^J_H,H_ = 6.3, 1 H, *cis*-CH_3_); 1.44 (s, 9 H, *t*-Bu-CH_3_); 1.47 (s, 9 H, *t*-Bu-CH_3_); 2.21–2.40 (m, 1 H, CH_2_); 2.67–2.82 (m, 1 H, CH_2_); 3.78–3.99 (m, 2.33 H, CH_2_ + *cis*-CH); 4.06 (dt, ^3^J_H,H_ = 4.1 Hz,^ 3^J_H,H_ = 8.5, 0.66 H, *trans*-CH); 4.94 (bs, 0.34 H, *cis*-NH); 5.13 (bs, 0.66, *trans*-NH). ^13^C NMR (CDCl_3_): δ 13.3 (+, 0.33 C, *cis*-CH_3_); 15.2 (+, 0.66 C, *trans*-CH_3_); 26.9 (+, 2 C, *trans*- *t*-Bu-CH_3_); 26.9 (+, 1 C, *cis*- *t*-Bu-CH_3_); 27.3 (+, 2 C, *trans*- *t*-Bu-CH_3_); 27.3 (+, 1 C, *cis*- *t*-Bu-CH_3_); 34.1 (−, 0.66 C, *trans*-CH_2_); 36.1 (−, 0.33 C, *cis*-CH_2_); 65.1 (−, 0.33 C, *cis*-OCH_2_); 65.8 (−, 0.66 C, *trans*-OCH_2_); 66.2 (C_quat_, 0.33 C, *cis*-NHC); 67.5 (C_quat_, 0.66 C, *trans*-NHC); 78.9 (C_quat_, 1 C, *t*-Bu-C); 79.9 (C_quat_, 1 C, *t*-Bu-C); 80.7 (+, 0.33 C, *cis*-CH); 81.0 (+, 0.66 C, *trans*-CH); 153.8 (C_quat_, 0.66 C, *trans*-NHCO); 154.1 (C_quat_, 0.33 C, *cis*-NHCO); 169.4 (C_quat_, 0.66 C, COO); 169.6 (C_quat_, 0.33 C, COO). IR (neat) [cm^−1^]: 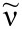 = 2973, 2361, 1705, 1489, 1369, 1247, 1158, 1073, 955, 849. CI-MS (NH_3_): *m/z* (%) = 202.1 (25) [MH^+^ − Boc], 246.1 (40) [MH^+^ − C_4_H_8_], 263.1 (34) [MNH_4_^+^ − C_4_H_8_], 302.1 (100) [MH^+^], 319.1 (4) [MNH_4_^+^]. HR-MS (FAB, MeOH/DCM/NBA): [MH^+^] calcd. for C_15_H_28_NO_5_ 302.1967; found 302.1966. MF C_15_H_27_NO_5_. MW 301.38.

### *tert*-Butyl 3-(*tert*-butoxycarbonylamino)-2-(prop-1-en-2-yl)tetrahydrofuran-3-carboxylate (**16**)

The synthesis followed GP 1 using [3-(*tert*-butoxycarbonylamino)-4-*tert*-butoxy-4-oxobutyl]dimethylsulfonium iodide (**1**, 2.39 g, 5.35 mmol, 1.5 equiv), caesium hydroxide (802 mg, 5.35 mmol, 1.5 equiv) and methacrylaldehyde (**8**, 294 µl, 3.57 mmol, 1 equiv). The product was purified with a 80:20 mixture of PE:diethyl ether (*R_f_* = 0.22) to give **16** as a colourless oil in 18% yield (210 mg, 0.64 mmol). ^1^H NMR (CDCl_3_): δ 1.37 (s, 9 H, *t*-Bu-CH_3_); 1.40 (s, 9 H, *t*-Bu-CH_3_); 1.69 (s, 3 H, CH_3_); 2.35–2.53 (m, 1 H, CH_2_); 2.57–2.71 (m, 1 H, CH_2_); 4.00 (q, ^3^J_H,H_ = 8.1 Hz, 1 H, OCH_2_); 4.12 (dt, ^3^J_H,H_ = 4.5 Hz, ^3^J_H,H_ = 8.4 Hz, 1 H, OCH_2_); 4.31 (s, 1 H, CH); 4.86 (s, 1 H, =CH_2_); 5.04 (s, 1 H, =CH_2_); 5.32 (bs, 1 H, NH). ^13^C NMR (CDCl_3_): δ 18.5 (+, 1 C, CH_3_); 26.8 (+, 3 C, *t*-Bu-CH_3_); 27.3 (+, 3 C, *t*-Bu-CH_3_); 34.8 (−, 1 C, CH_2_); 66.4 (C_quat_, 1 C, NHC); 67.5 (−, 1 C, OCH_2_); 81.2 (C_quat_, 2 C, *t*-Bu-C); 85.5 (+, 1 C, CH); 112.2 (−, 1 C, =CH_2_); 139.7 (C_quat_, 1 C, C=); 153.4 (C_quat_, 1 C, NHCO); 169.4 (C_quat_, 1 C, COO). IR (neat) [cm^−1^]: 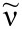 = 3409, 3059, 2063, 1614, 1483, 1335, 1242, 1133, 1098, 1055, 887, 823, 708. CI-MS (CI, NH_3_): *m/z* (%) = 228.1 (28) [MH^+^ − Boc], 272.1 (41) [MH^+^ − C_4_H_8_], 289.1 (50) [MNH_4_^+^ − C_4_H_8_], 328.1 (100) [MH^+^], 345.1 (8) [MNH_4_^+^]. HR-MS (FAB, MeOH/glycerol): [M^+^] calcd. for C_17_H_29_NO_5_ 327.2046; found 327.2043. MF C_17_H_29_NO_5_. MW 327.42.

## Supporting Information

File 1 NMR spectra of compounds **12**–**16**

File 2 CIF file of compound **15**
