# Supplementary material for: Synthesis of new Cα-tetrasubstituted α-amino acids
Source: Beilstein J Org Chem. 2009 Feb 18;5:5. doi: 10.3762/bjoc.5.5 (PMC2649440; doi:10.3762/bjoc.5.5)
Supplement: File 1 — NMR spectra of compounds 12–16 [file Beilstein_J_Org_Chem-05-05-s001.doc]

**-Supporting Information-**

Copies of NMR spectra

**Synthesis of new C^α^-tetrasubstituted α-amino acids**

*Andreas A. Grauer and Burkhard König**

Address: Institute for Organic Chemistry, University of Regensburg, Universtätsstrasse 31, 93040 Regensburg, Germany

Email: Burkhard König - burkhard.koenig@chemie.uni-regensburg.de

* Corresponding author


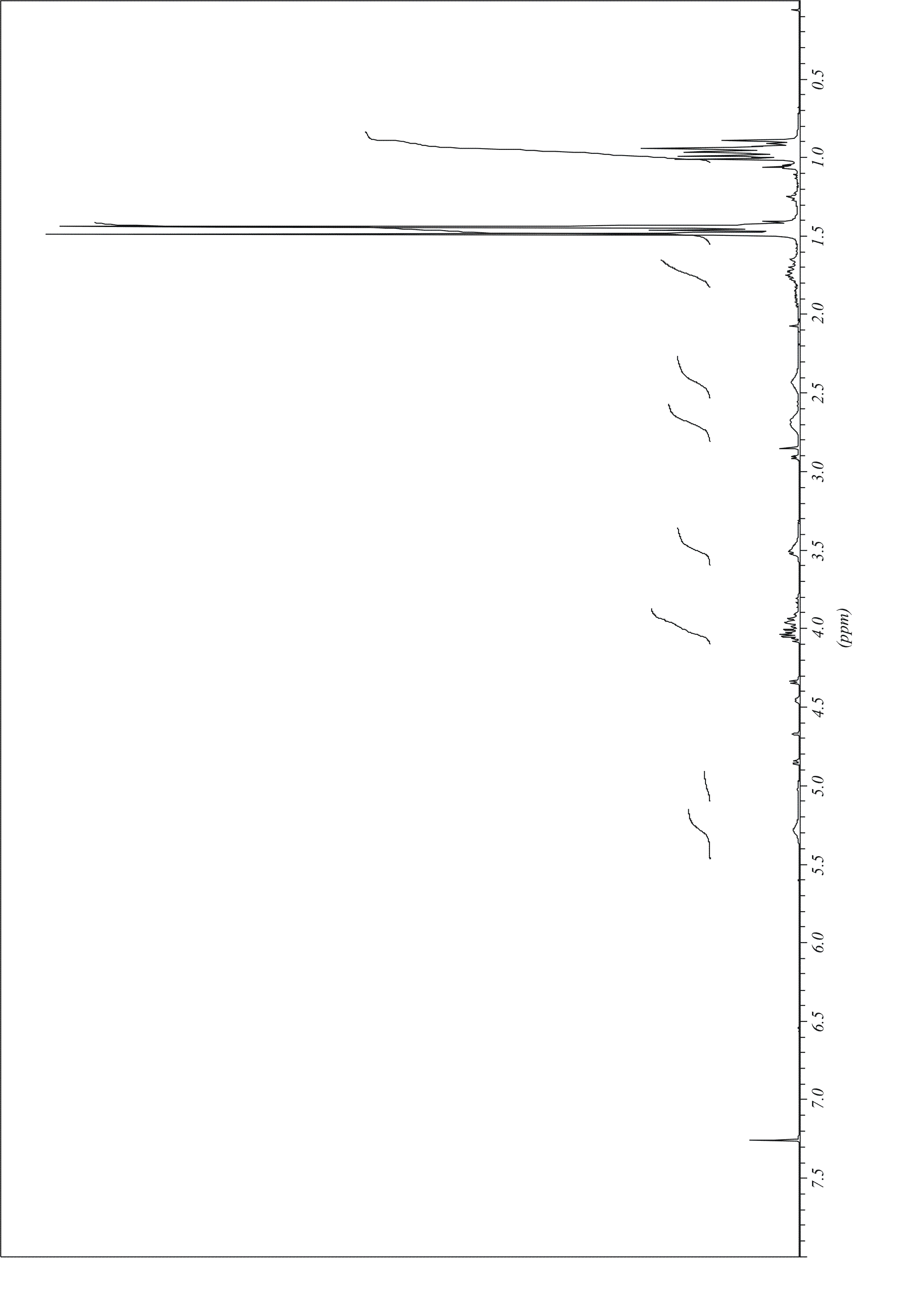

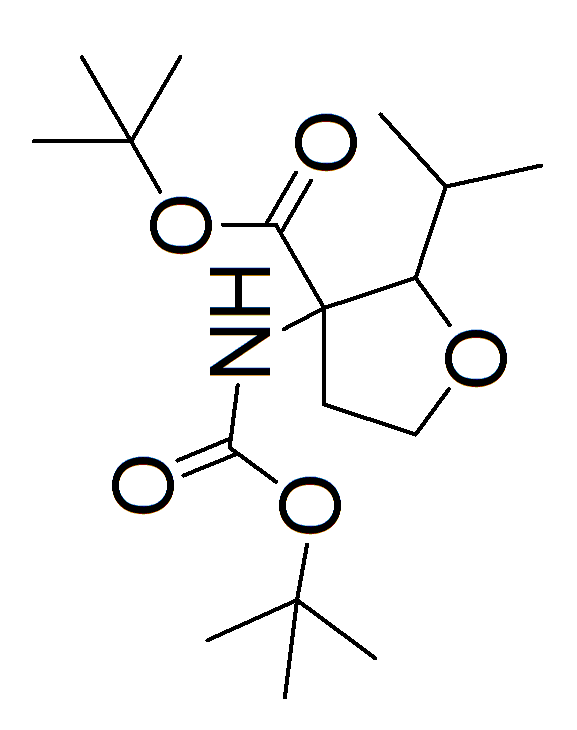
1H NMR of *tert*-butyl 3-(*tert*-butoxycarbonylamino)-2-isopropyltetrahydrofuran-3-carboxylate (**12**; CDCl_3_, 300 MHz):

impurities

impurities


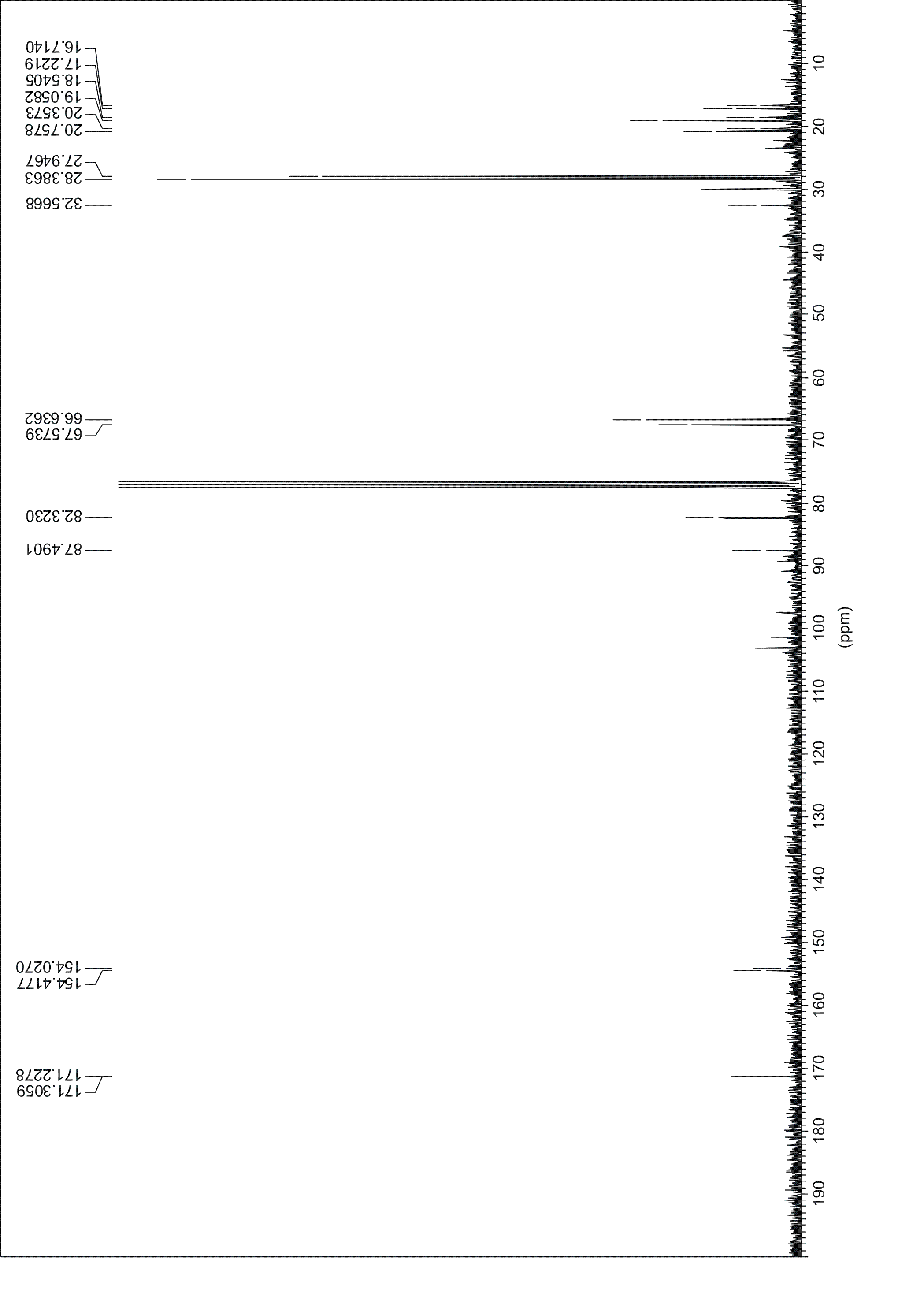
13C NMR of *tert*-butyl 3-(*tert*-butoxycarbonylamino)-2-isopropyltetrahydrofuran-3-carboxylate (**12**; CDCl_3_
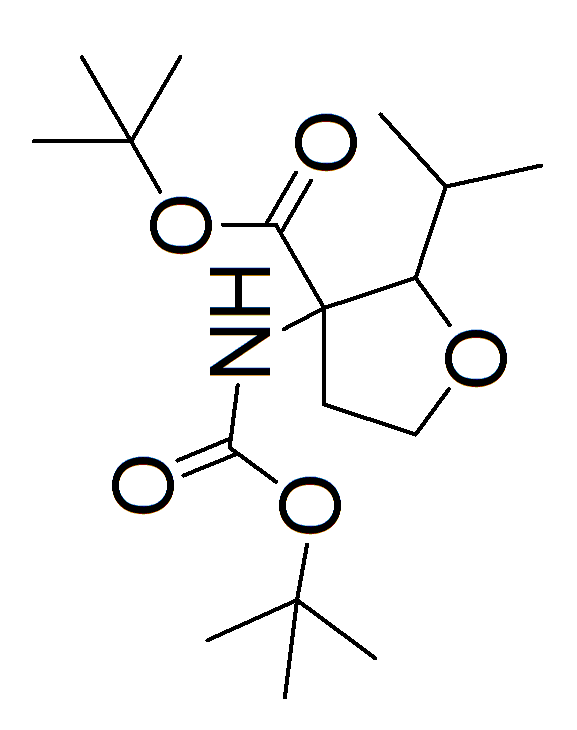
, 75 MHz):

^1^H NMR of *tert*-butyl 3-(*tert*-butoxycarbonylamino)-2-isobutyltetrahydrofuran-3-carboxylate (**13**; CDCl_3_
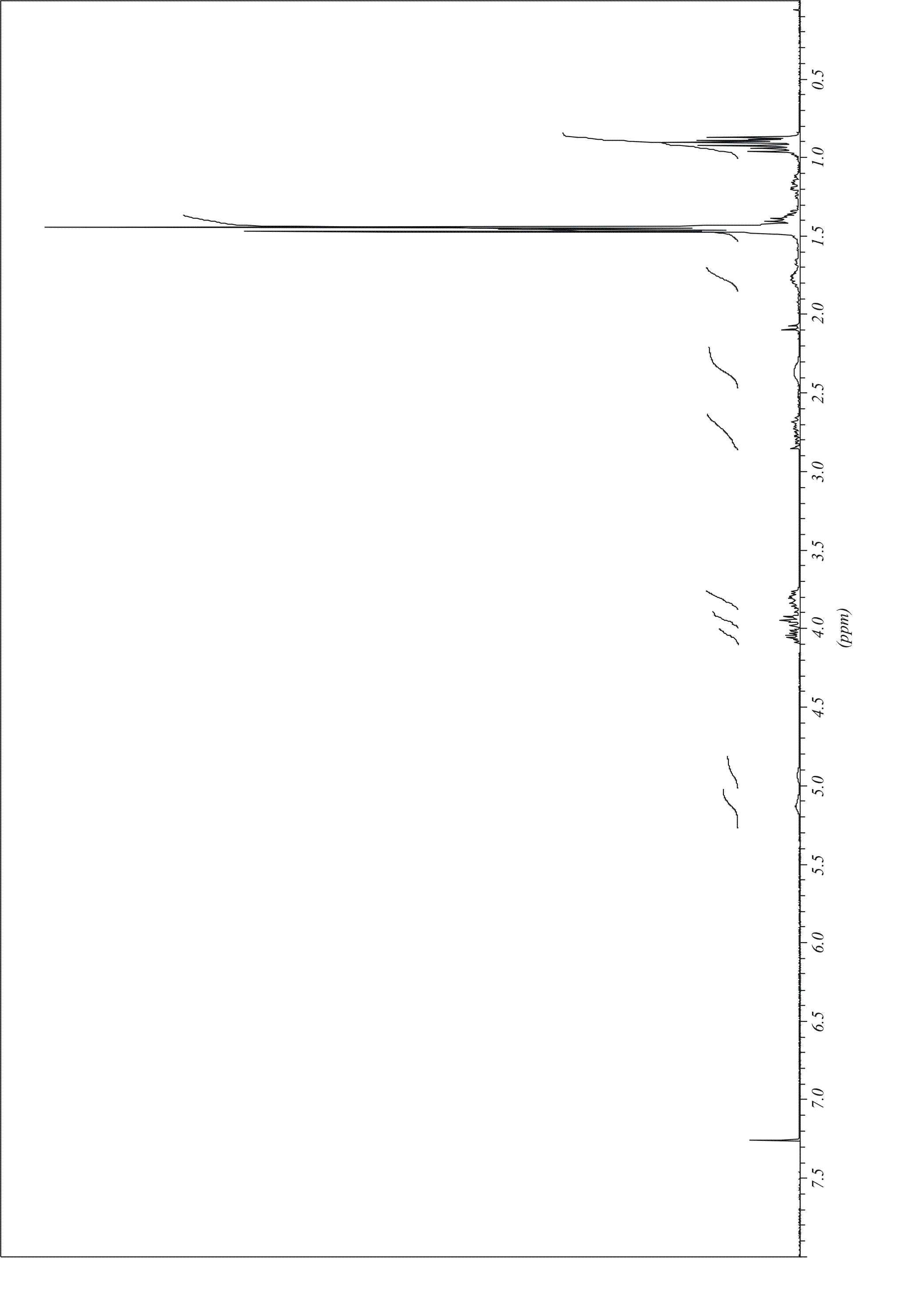

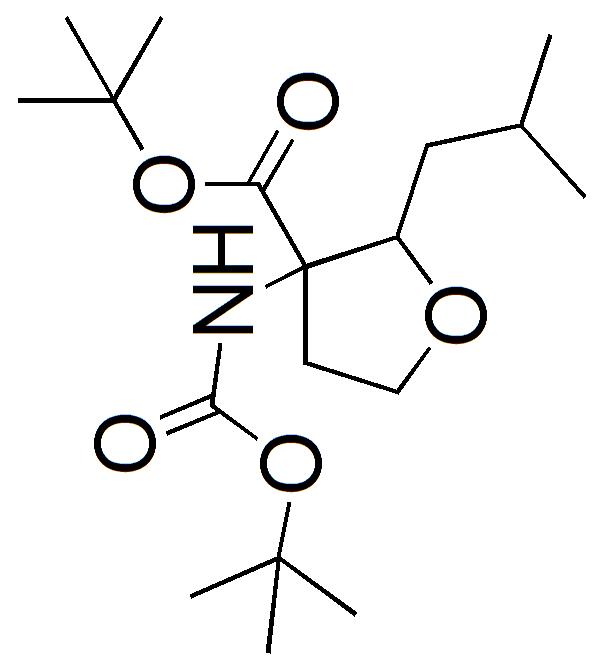
, 300 MHz):


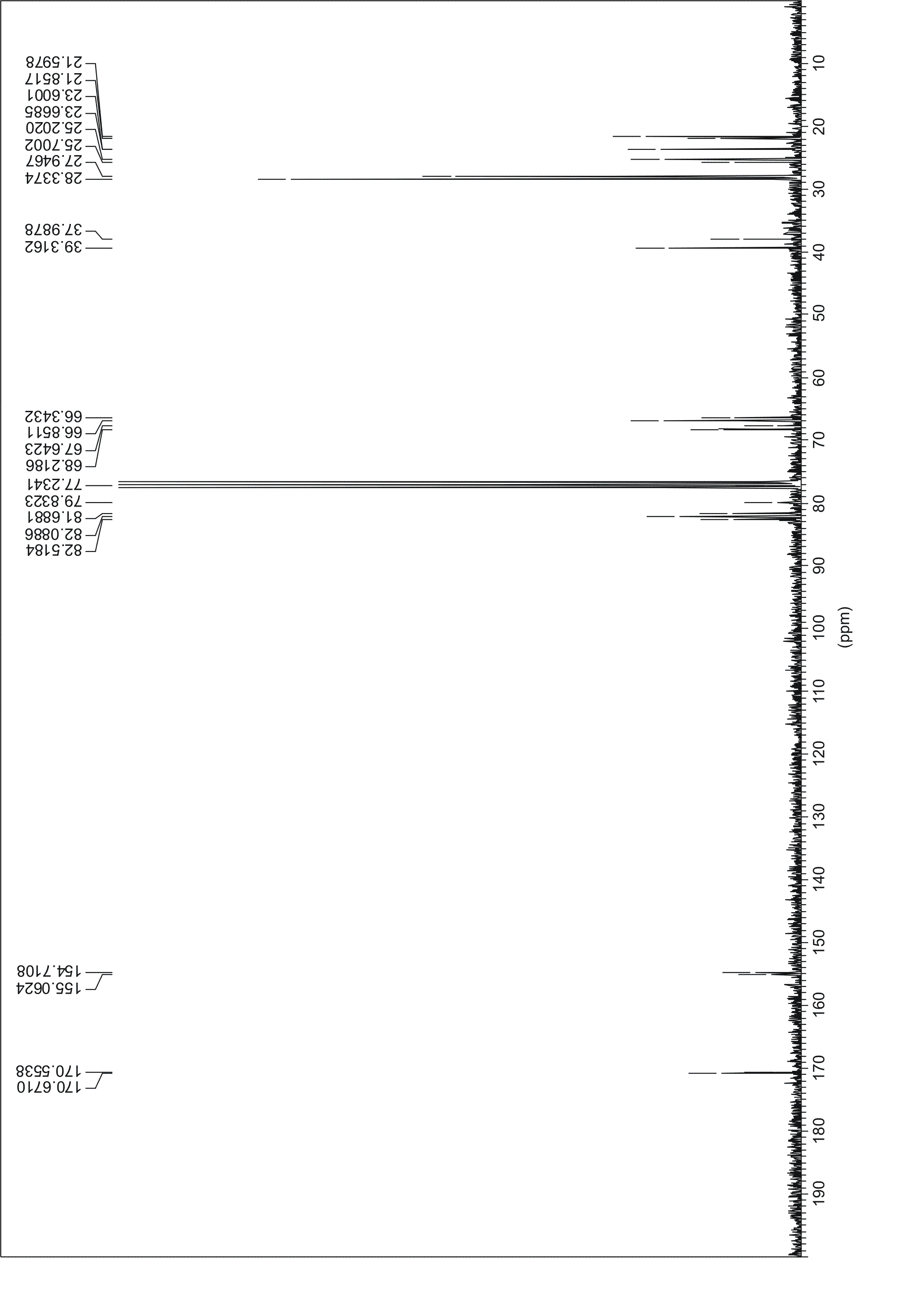

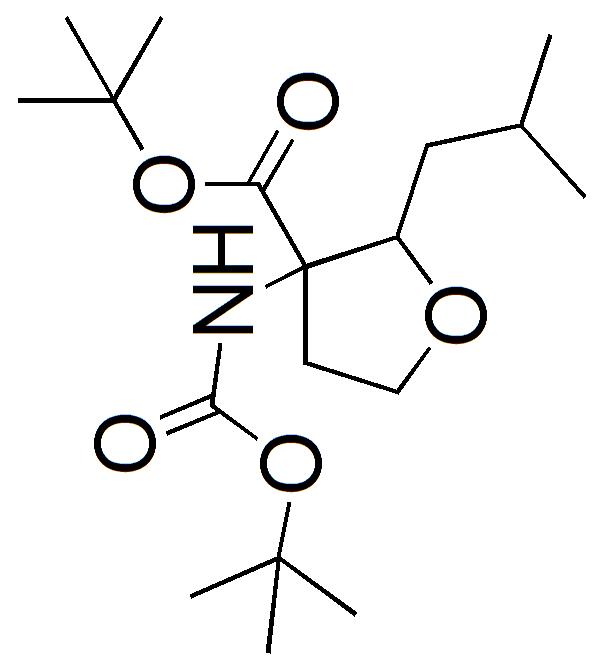
^13^C NMR of *tert*-butyl 3-(*tert*-butoxycarbonylamino)-2-isobutyltetrahydrofuran-3-carboxylate (**13**; CDCl_3_, 75 MHz):


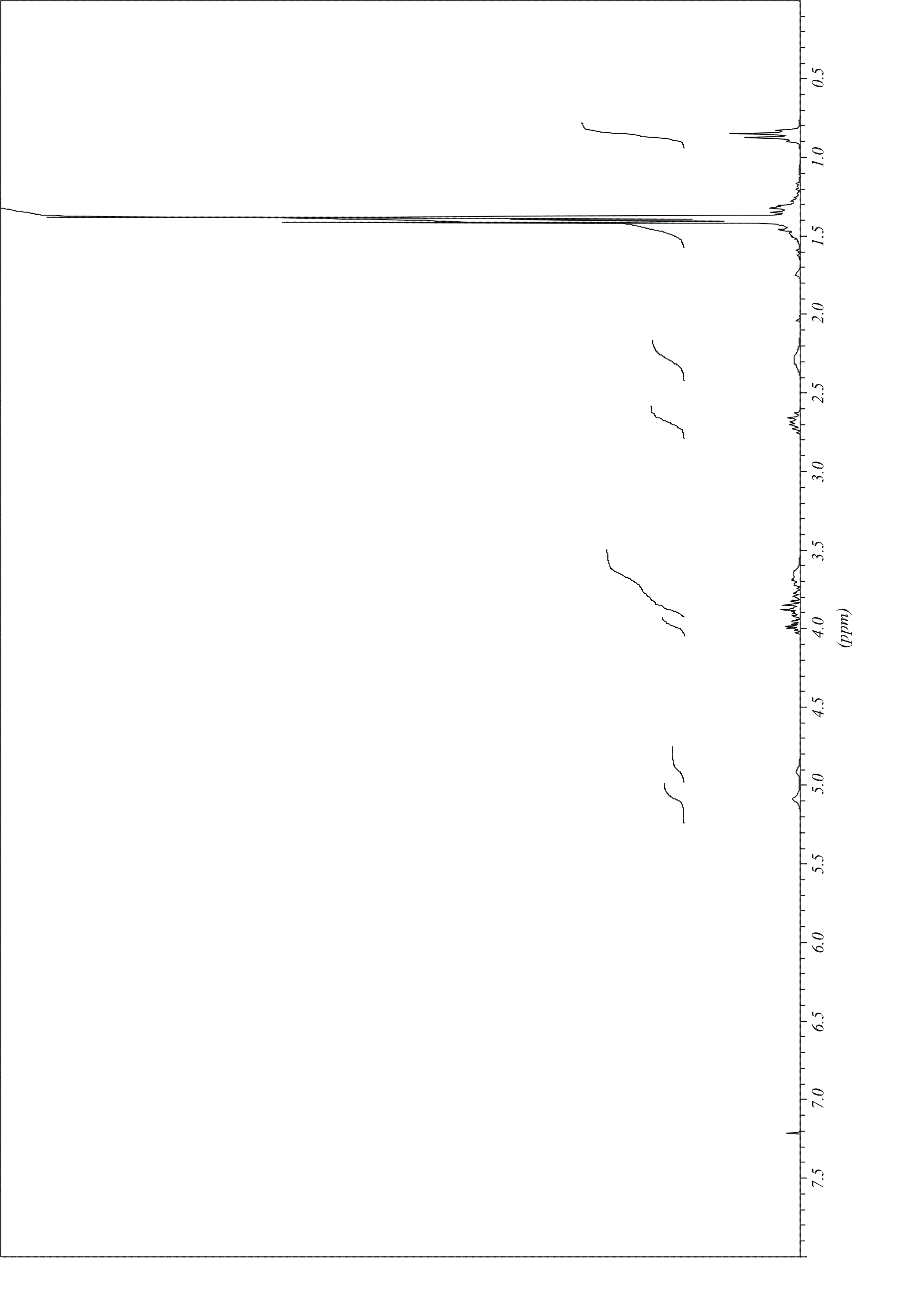
1H NMR of *tert*-butyl 3-(*tert*-butoxycarbonylamino)-2-propyltetrahydrofuran-3-carboxylate (**14**; CDCl_3_
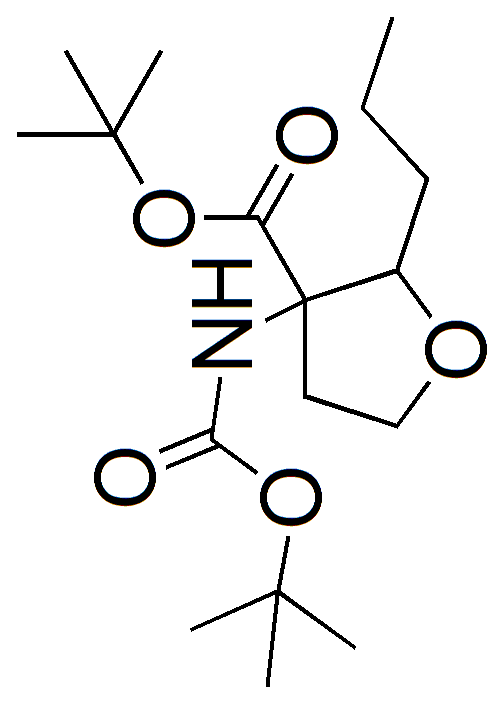
, 300 MHz):


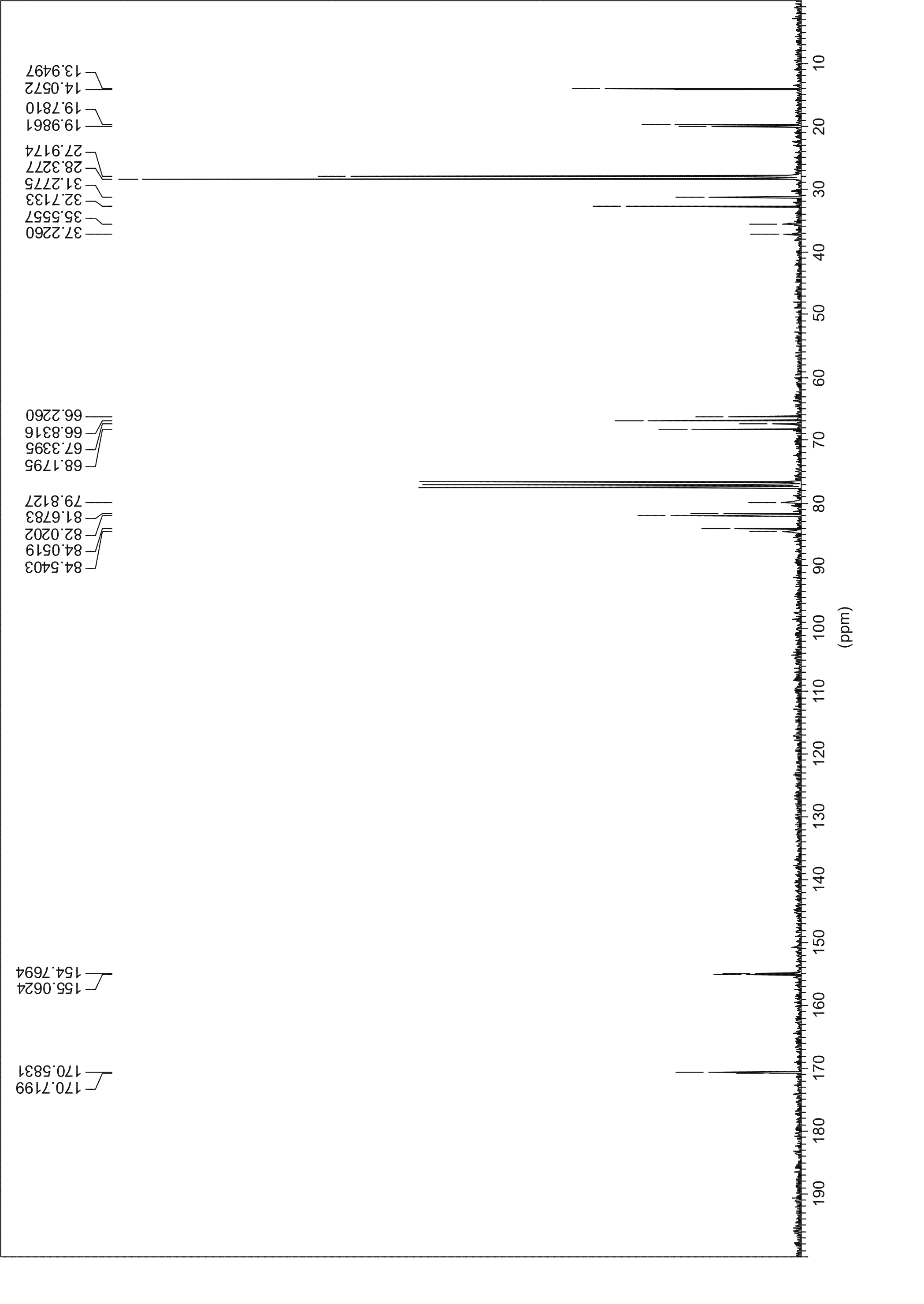
13C NMR of *tert*-butyl 3-(*tert*-butoxycarbonylamino)-2-propyltetrahydrofuran-3-carboxylate (**14**; CDCl_3_
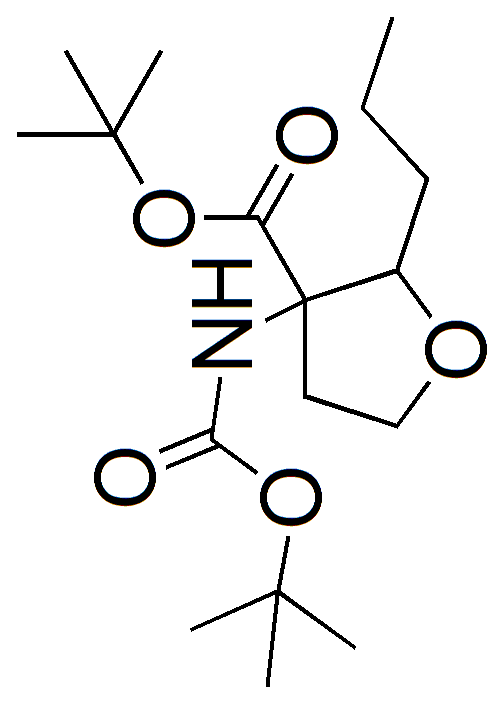
, 75 MHz):


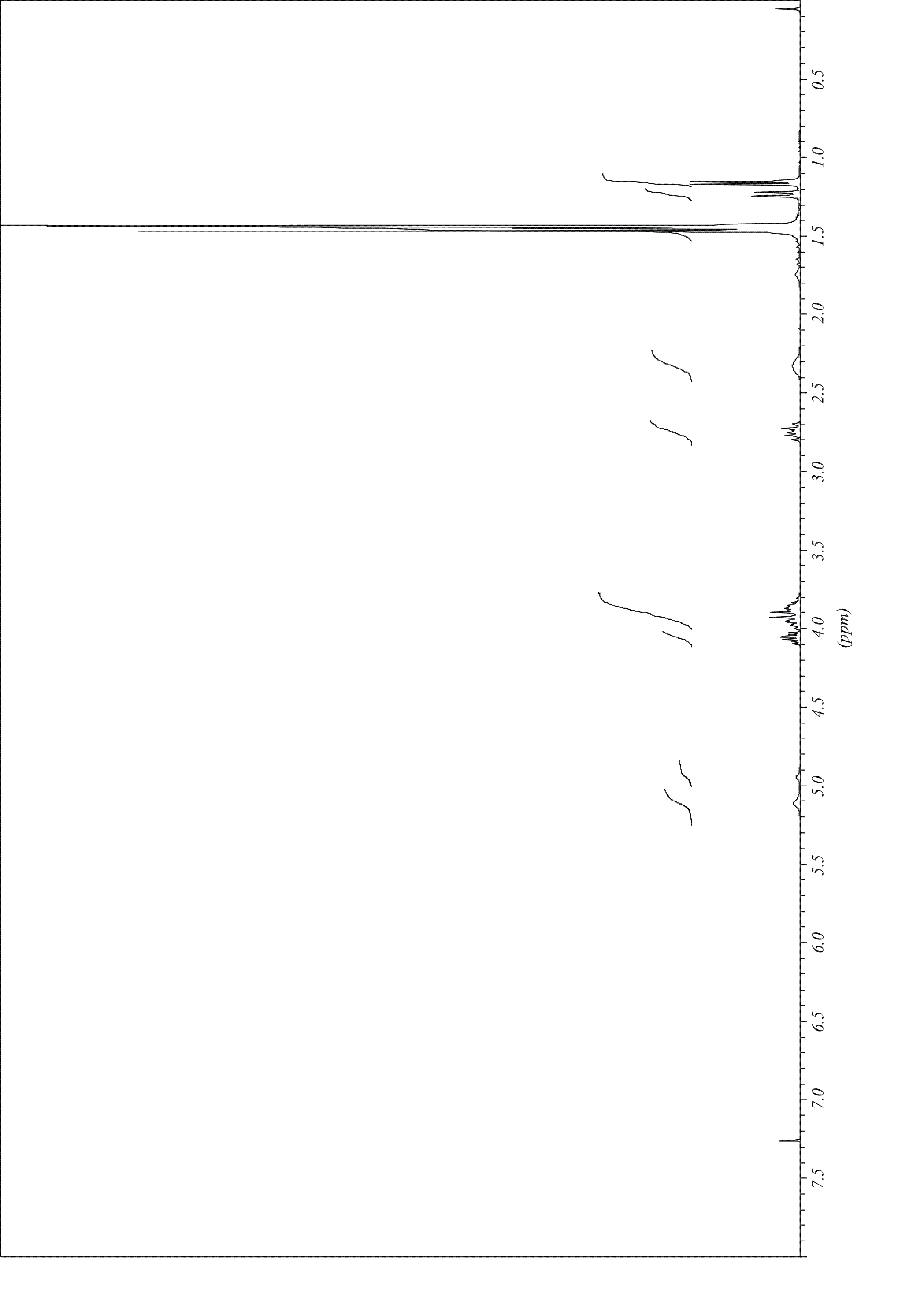

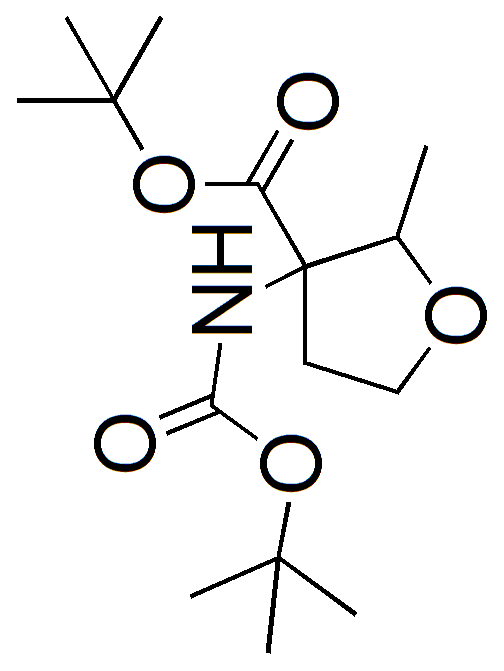
^1^H NMR of *tert*-butyl 3-(*tert*-butoxycarbonylamino)-2-methyltetrahydrofuran-3-carboxylate (**15**; CDCl_3_, 300 MHz):


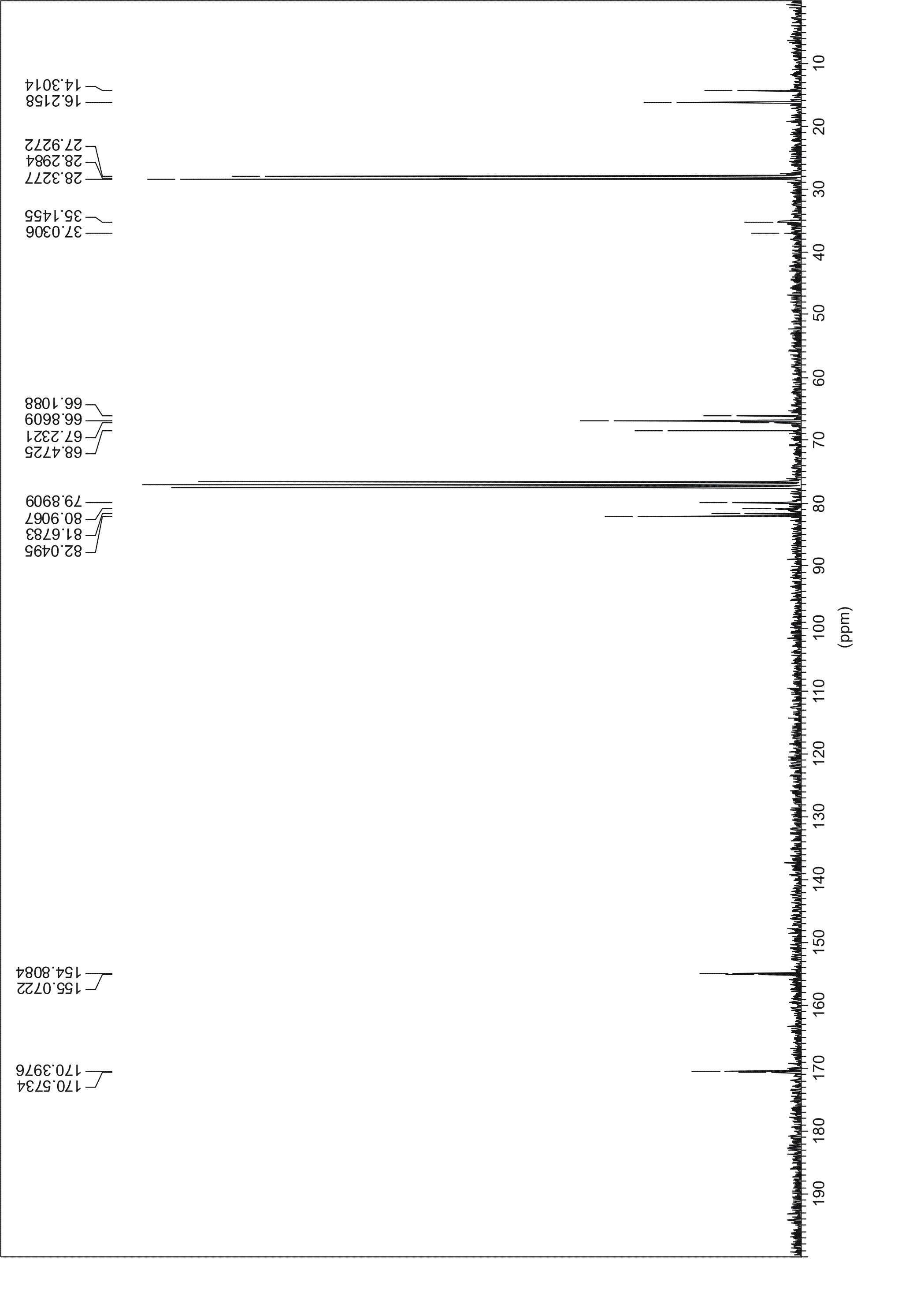

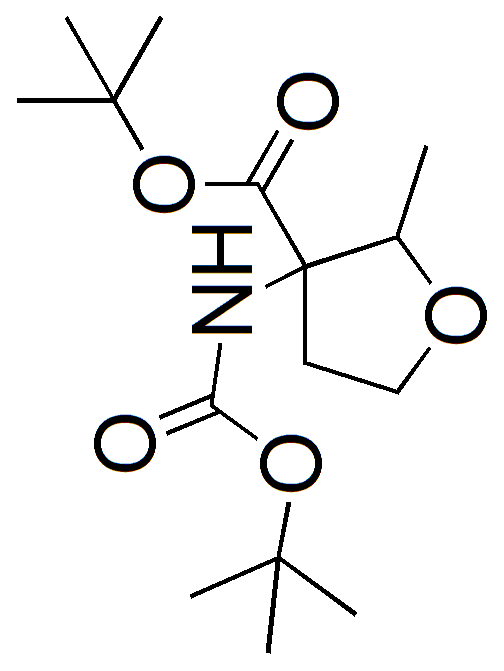
^13^C NMR of *tert*-butyl 3-(*tert*-butoxycarbonylamino)-2-methyltetrahydrofuran-3-carboxylate (**15**; CDCl_3_, 75 MHz):


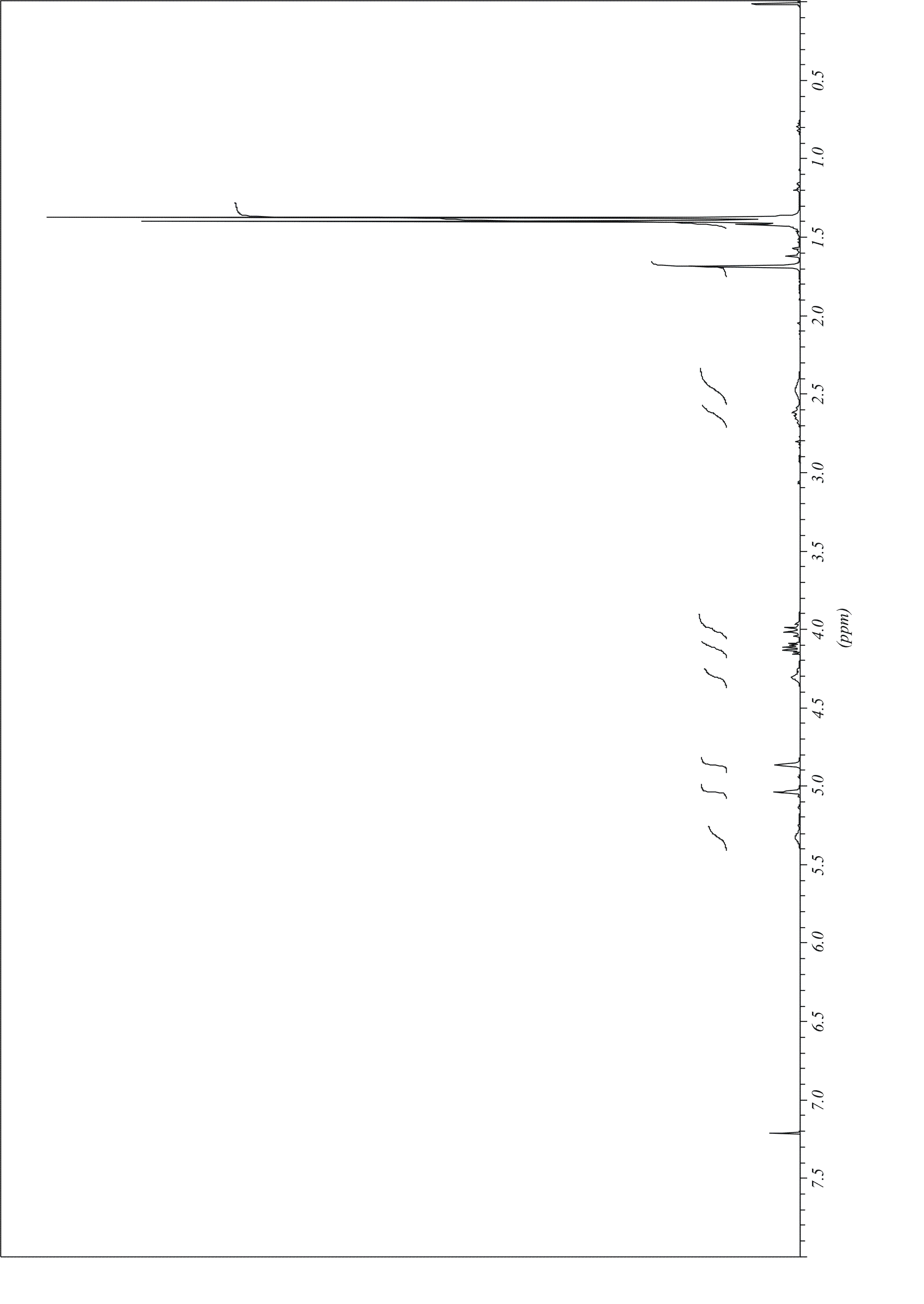
1H NMR of *tert*-butyl 3-(*tert*-butoxycarbonylamino)-2-(prop-1-en-2-yl)tetrahydrofuran-3-carboxylate (**16**; CDCl_3_
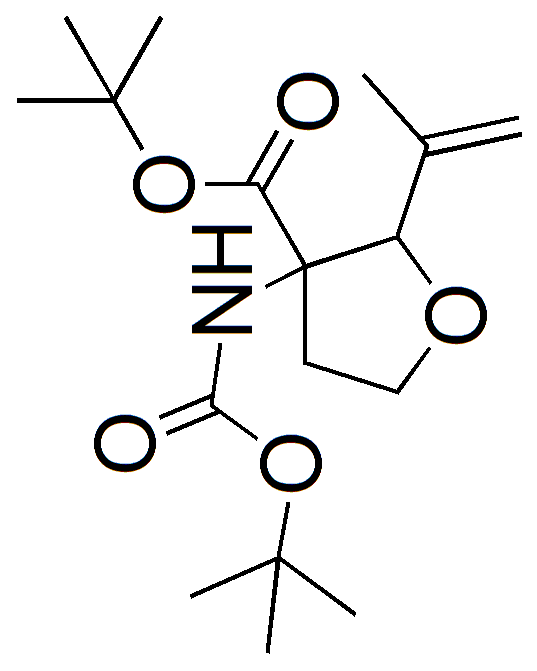
, 300 MHz):


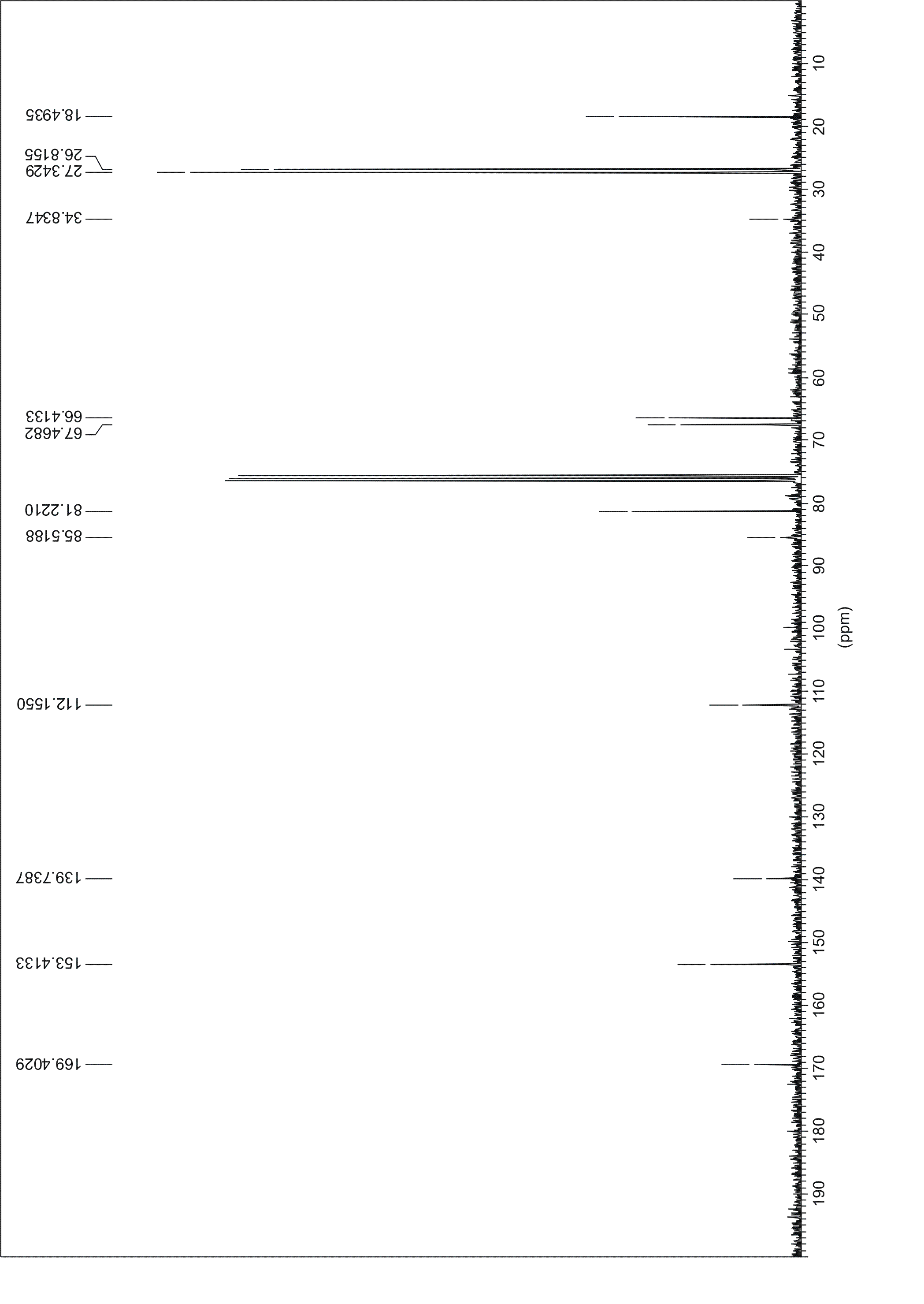

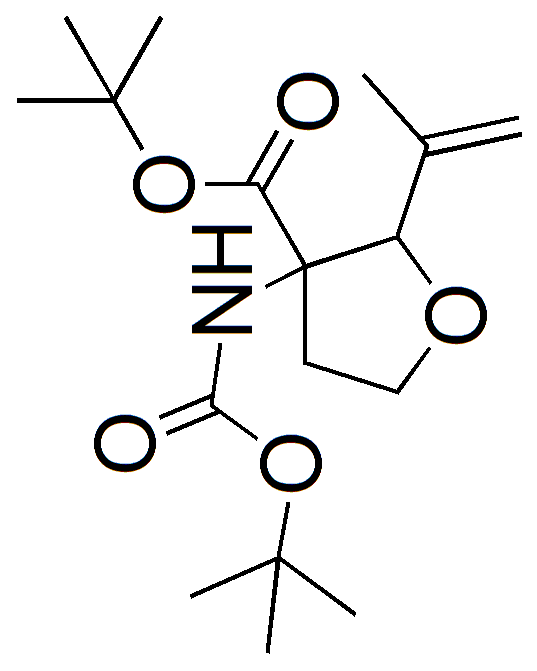
^13^C NMR of *tert*-butyl 3-(*tert*-butoxycarbonylamino)-2-(prop-1-en-2-yl)tetrahydrofuran-3-carboxylate (**16**; CDCl_3_, 75 MHz):

NOESY-spectra of
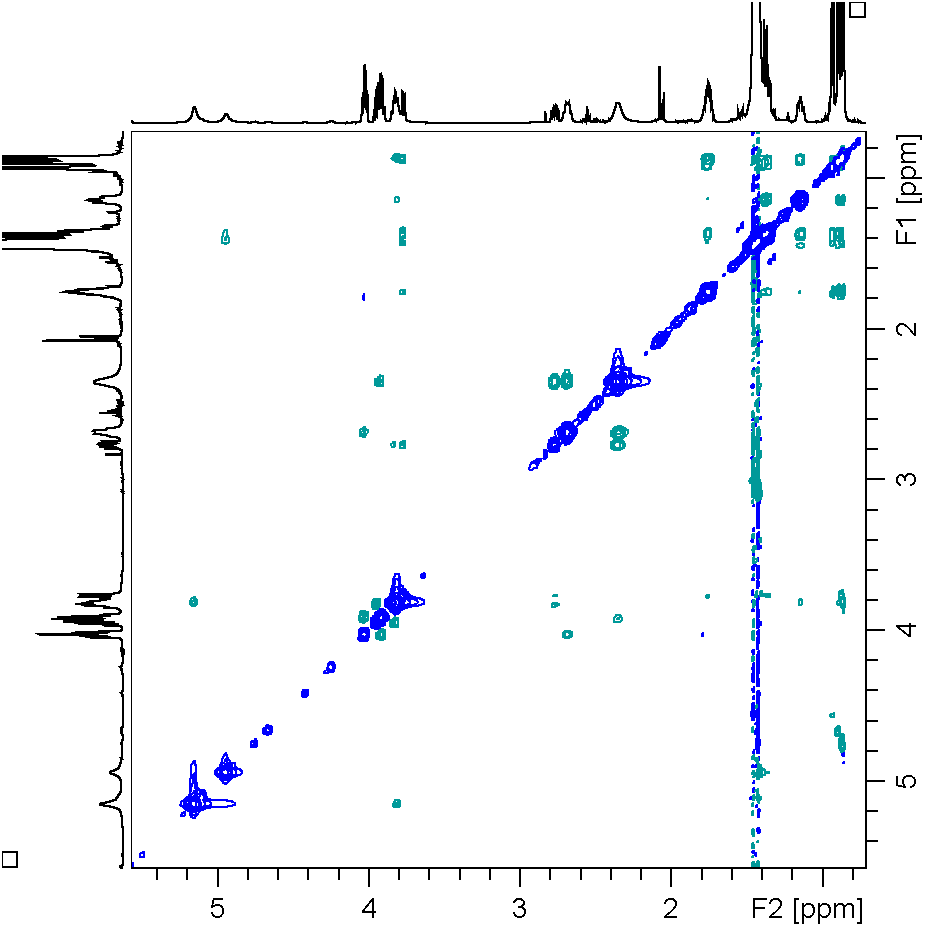
**13** (CDCl3, 600 MHz):

trans / cis

NH

THF-CH

NH

trans / cis

THF-CH

NH / THF-CH cross peak of the *trans*-product

Absence of the NH / THF-CH cross peak for the *cis*-product
